# Low-Cost Ultrasonic Distance Sensor Arrays with Networked Error Correction

**DOI:** 10.3390/s130911818

**Published:** 2013-09-05

**Authors:** Hongjun Dai, Shulin Zhao, Zhiping Jia, Tianzhou Chen

**Affiliations:** 1 Department of Computer Science and Technology, Shandong University, No. 1500, Shunhua Road, Jinan 276023, China; E-Mails: zhaoshulin.cn@gmail.com (S.Z.); jzp@sdu.edu.cn (Z.J.); 2 Department of Computer Science and Technology, Zhejiang University, No. 38, Zheda Road, Hangzhou 310027, China; E-Mail: tzchen@zju.edu.cn

**Keywords:** ultrasonic distance sensor, networked error correction, confidence distance, maximum likelihood method, neighbor discovery, correlation coefficient

## Abstract

Distance has been one of the basic factors in manufacturing and control fields, and ultrasonic distance sensors have been widely used as a low-cost measuring tool. However, the propagation of ultrasonic waves is greatly affected by environmental factors such as temperature, humidity and atmospheric pressure. In order to solve the problem of inaccurate measurement, which is significant within industry, this paper presents a novel ultrasonic distance sensor model using networked error correction (NEC) trained on experimental data. This is more accurate than other existing approaches because it uses information from indirect association with neighboring sensors, which has not been considered before. The NEC technique, focusing on optimization of the relationship of the topological structure of sensor arrays, is implemented for the compensation of erroneous measurements caused by the environment. We apply the maximum likelihood method to determine the optimal fusion data set and use a neighbor discovery algorithm to identify neighbor nodes at the top speed. Furthermore, we adopt the NEC optimization algorithm, which takes full advantage of the correlation coefficients for neighbor sensors. The experimental results demonstrate that the ranging errors of the NEC system are within 2.20%; furthermore, the mean absolute percentage error is reduced to 0.01% after three iterations of this method, which means that the proposed method performs extremely well. The optimized method of distance measurement we propose, with the capability of NEC, would bring a significant advantage for intelligent industrial automation.

## 1. Introduction

Distance is one of the most basic factors in manufacturing and control fields. It is used for local positioning, object identification, automation control, human-computer interaction, and so on [1,2]. In order to measure distance, ultrasonic distance sensors (UDS) have been widely used as a low-cost solution [3], and large numbers of them have been set up intensively as sensor arrays [4]. For example, in the production lines of assembly workshops—to ensure the size of products, and in parking guidance systems for shopping malls—to detect and classify automobiles. However, the propagation of ultrasonic waves is greatly affected by the environmental factors such as temperature, humidity, and atmospheric pressure [5]. Many traditional methods [6] have been employed to compensate for errors introduced by the sensor design. Furthermore, with the development of intelligent industrial automation, sensors have been networked to interchange data, and can easily gain supplementary information from neighboring nodes. In combination with these features, this paper proposed a novel design of sensor arrays that can correct its own errors with information from the network.

**Figure 1.**
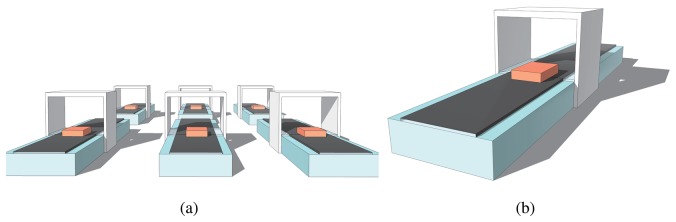
Scenario of production lines. (**a**) Parallel lines in a workshop. (**b**) One line in a workshop.

As shown in Figure 1, taking the scenario of production lines as an example, there are many parallel lines in a workshop. A number of UDS are set up in each line to measure the size of the semi-finished products, and the measured values are passed to a data center (DC) to judge the quality through the network. The measured values are unavoidably imprecise, because these sensors are in different micro-environments. To solve this issue, each UDS can be equipped with the temperature and humidity sensors to correct errors caused by the environment. However, this requires a greater hardware cost, more redundant circuit designs, and more complex compensation algorithms. Nevertheless, with the information-based development of modern industry, the data from the sensors can be gathered by the DC for centralized interactive control. A hardware upgrade of UDS can be avoided—decreasing the total cost—if an effective method of DC data analysis and subsequent error correction is carried out.

The most basic method of UDS is using a simple pulse echo sensor, which acts as both transmitter and receiver. An echo is detected by the receiver after the transmitted pulse propagates outwards. The distance between the transmitter and receiver is obtained by counting the elapsed time from the start of the transmission to the end of the receipt, defined as the time-of-flight (TOF) [7]. Given that ultrasonic speed is a fixed value in a certain environment, the distance can be calculated by the following Equation:

(1) $$d_{s}=\frac{v_{s}\times \Delta T}{2}$$

where *d_s_* is the measured distance, *v_s_* is the ultrasonic propagation velocity in the medium, and Δ*T* is the signal TOF. Then, the precision of *d_s_* depends on the precision of *v_s_* and Δ*T*. In particular, the distance between the sensor and the floor is a fixed value *d*_0_ if nothing is measured. Therefore, there is a zero mode (Z-MODE) in the device meaning that, irrespective of the measured value from the UDS, the distance is regarded as *d*_0_ without any definite errors.

The fact that the industrial environments of sensors are non-ideal implies that it is sometimes difficult to detect the real size of the semi-manufactured products. In order to improve the detection process, many mathematical methods have been used, closely followed by the use of UDS growing in an exponential manner since the 1980s [8]. On one hand, using a comparative and collaborative approach is beneficial to multi-analysis and produces compelling results. In [9], it not only presents an optimized ultrasound-driven method of incorporation of the amplitude modulation and phase modulation of the transmit-receive technique, but also proposes two optional algorithms to achieve TOF calculations, according to different signal-to-noise ratio situations—named the zero-crossing tracking and time-shifted superposition methods. In [10], it applies the discrete extended Kalman filter to the acquired ultrasonic signal, in order to accurately estimate the shape factors of the echo envelope as well as to locate its onset; it is also possible to ensure reduced bias and uncertainty in critical TOF measurements. In [11], it introduces the received ultrasonic wave peak time sequences of two slightly different frequencies, and the experimental results demonstrate that the relative TOF can be accurately identified with much better resolution than the wave period. On the other hand, modeling based on the network topology of sensor arrays has already been applied in some modern industrial cases.

As mentioned above, in some of modern industrial cases, these devices have formed a network with wired or wireless methods passing data to a DC. Furthermore, many data mining and mathematical and statistical algorithms have been used for data analysis. A model using data mining algorithms which then processes data based on the same model has been widely employed in industrial fields to gain accurate measurements. In [12], it uses the statistics of the relation of selection and theoretical variances, the simultaneous operative testing of mathematical expectation, and the variance of errors in the one-dimensional measurements. Then in [13], an approach to characterize the model prediction errors using a Gaussian mixture model is proposed, which mainly consists of choosing the relevant variables to form the error data, optimizing the number of Gaussian components required for the error data modeling, and fitting the Gaussian mixture parameters with an expectation-maximization algorithm. In [14], it proposes a two-mode adaptive fuzzy controller with an approximation error estimator. In the learning mode, the controller employs some modified adaptive laws to tune the fuzzy system parameters and an approximation error estimator to compensate for the inherent approximation error; in the operating mode, the fuzzy system parameters are fixed—only the estimator is updated online. These methods have been effectively used for data analysis, so they can also be incorporated into networked UDS systems for error correction. In general terms, previous studies have suggested that using methods that focus on optimization of a single UDS device can improve the performance of distance measurement to some extent, however, contradictions among the accuracy, hardware complexity, data update rate and measurable range still exist.

In this study, based on the fact that UDS devices have been organized as a network to gather real-time data into a DC, we focus on the mathematical methods to calculate the precise correction value by analysis of the data correlation coefficients *via* networked error correction (NEC). The main features of the method are briefly described in this paragraph, where the model of NEC and the analysis of the data correlation coefficients are also presented. Field tests are implemented in this study, because they are more reliable and more likely to reveal potential problems. To the best of our knowledge, we identify for the first time that a method using network topology information and leveraging recent advances in optimization would be a more useful technology for distance measurement.

Over all, the contributions of this paper are summarized as the followings:

- We have modeled the NEC system on the optimization of the relationship of the topological structure of the sensor arrays, and developed a Two-Step Error Correction Process (TSECP) based on the relations between neighboring nodes.
  – An abstract model is set up to illustrate the ability of the compensation for error measurements caused by the environments.
  – TSECP is described with the theory of fusion set. To compare with the previous NEC methods, we have analyzed the design comparisons and the system improvements.
- We have designed the UDS with the maximum likelihood method, neighbor discovery discovery algorithm and the NEC optimization algorithm.
  – We apply the maximum likelihood method to determine the optimal fusion data set and use a neighbor discovery algorithm to identify neighbor nodes at the top speed.
  – We adopt the NEC optimization algorithm, take full advantage of the correlation coefficients for neighbor sensors.

The experimental results demonstrate that the NEC system has excellent anti-interference performance, and its ranging errors are within 2.2%. Furthermore, the mean absolute percentage error is reduced to 0.01% after three iterations of this method.

Because we take advantage of data correlation coefficients for the first time, the novel NEC model is more accurate than existing approaches. Its superiority over the former methods is obvious: instead of the solo node, all of the topological information of the sensor arrays is involved in error compensation. Observational evidence has frequently been linked to numerical error and propagation of the ultrasonic wave affected by the environment; however, there is little direct trial evidence to train and verify the exact equation of the two parameters. In view of the significant potential value of this issue, further investigation of the equation is warranted.

## 2. Related Work and Motivation

### 2.1. Distance Measurement by Ultrasonic Sensor Arrays

Recently, distance measurement based on UDS has increased significantly. In [15], according to the multi-sensor information fusion theory, it uses Kalman filter principle to deal with single ultrasonic sensor information and multi-ultrasonic sensor information fusion. The results show that multi-sensor distance measurement is more accurate and stable than single sensor distance measurement. In [16], it also proposes the experimental dimensionless curves/corresponding equations. Then, it presents the relative profile of calibrating coefficients and ambient temperature of the specific ultrasonic sensor to accelerate the field calibrating process. Obviously, it is unavailable before the formal stage measuring, so the calibrating accuracy with equation estimation is slightly inferior to this process.

Moveover, the performance of the UDS arrays system can be greatly improved by adopting data fusion technologies and self-configuring scheduling protocols. In [17], it presents a self-configuring protocol for time-slot allocation problem, under the constraint that no explicit communication is allowed in the sensor system. Simulations with two sensor geometries have demonstrated the effectiveness of the proposed protocol. In [18], it presents a novel approach toward the identification of odors/gases using game theory for feature selection. Considering that the merit of this approach lies in the fact that each dimension of the raw data gets adequate representation in the form of Shapley value assignment based upon its contribution to class separability. However, it can not be compared to a standard sample considering the lack of appropriate scaling of test samples.

Notably, the implementations of UDS arrays have obtained increasingly influence in the industrial world. In [19], based on the two-frequency continuous-wave phase-shift method, it has developed a high-resolution UDS system using vernier caliper phase meter, which can reduce the cost and complexity of the circuit elements, as well as decrease the noise disturbance induced from the high-frequency clock signal. In [20], it presents a localization system for Wireless Sensor Networks (WSN) based on ultrasonic TOF measurements. The mean error and standard deviation of the angle and distance measurements lead to a small mean localization error of 4.21 cm and a standard deviation of 0.57 cm. But there are also some disadvantages: ultrasonic consumes more energy, it has a lower range; and the speed of sound depends on the environmental temperature and humidity.

### 2.2. Networked Error Correction with Mathematical Statistics

NEC is the key stone for the implementation of UDS. In [21], it introduces the concept of co-integration, describing an equilibrium relationship among a set of time-varying variables, which can be utilized to represent the co-integrated relationship through an error correction model and examine the effect or local influence on the error correction variable. From another perspective, methods based on the topological structure relationship of components have been applied to data analysis. In [22], a forward error correction scheme for WSN has been developed to avoid retransmission. It not only saves energy but also extends the functionality and enables to handle “BURST ERRORS”. It is more effective because the energy expended for the retransmission in the case of the original correction scheme is saved when the new proposed error correction scheme is implemented. In [23], in order to address the fact that distance measurements are often corrupted by large errors which will lead to inaccurate position estimates without correction, it has proposed a scheme of joint node localization and error correction for the distance measurements of WSN. Compared with the centralized algorithms, decentralized methods are more robust and scalable. However, it does not studies the case that the position information of anchors are inaccurate. In [24], it has proposed an adaptive forward error correction algorithm for the best effort WSN. This solution does not require additional feedback channels, which makes it compatible with WSN. However, it has not justified the choice of link quality metric for this work and gained a deeper understanding of the contribution.

Moreover, mathematical statistics has been widely used for NEC. In [9], it presents a two-level neural network fed with the acquired samples of each echo received, employed for post-processing. A competitive layer has been used to determine which period of the echo waveform made the threshold detector fire; then, a multi-layer perception used to determine the actual echo position. These methods focus on the optimizations in the single UDS devices, which put extra pressures on the computation of the source-limited UDS device. In [25], it has proposed two estimators based on correlation parameters for the two key steps of a practical distributed source coding scheme: first, the computation of the side-information at the receiver side; second, the estimation of the required number of bits to compress the readings in order to guarantee a certain symbol error probability. This method allows to reduce the number of transmitted bits and hence reduces the energy consumption. In [26], it firstly studies the error between the value obtained from the sample data of all or a part of nodes, and carries out algorithms to assign confidence levels to child nodes of the root on the basis of their corresponding confidence levels. The experimental results illustrate that this scheme can save energy extremely. However, it has not analyzed the error and confidence level allocation when the beginning time of each node to sample data is asynchronous.

### 2.3. Motivation

The observations above demonstrate that measured values are unavoidably imprecise, because of the instability of the environment. This motivates us to propose an approach of minimizing the error between the measured response and the desired response. This study provides a potential solution based on NEC for the reliable and low-cost distance-measurement applications by using the information from neighboring sensors—which has not been considered before. This also has the potential to shed light on the automation fields.

## 3. Process of Networked Error Correction

### 3.1. Basic Architecture

**Figure 2.**
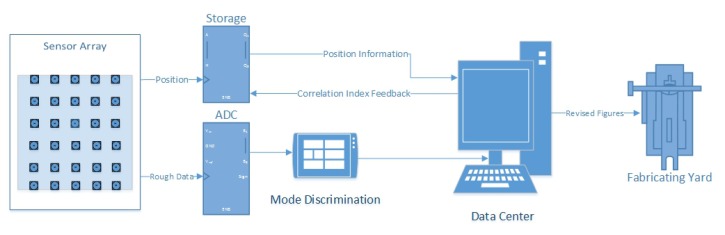
Block diagram of the basic architecture.

The basic architecture of networked sensors is shown in Figure 2. Each node has a fixed position and measures the distance independently. All of them constantly pass the measured data to the DC. Besides positional information, two other aspects are considered with changes in time. First, at some time point *t* all of the nodes measure the raw data without any correction hardware (temperature or humidity sensors). If a node is in Z-MODE, its value can be compared to the precise value *d*_0_, and the error of the node can be found by a simple subtraction. Second, in a time interval, *t* ∈ [*t*_1_, *t*_2_], each node measures an independent series of data points (because of the different objects measured). There is no distinctive function for a node; however, for the nodes in their fixed positions, the errors caused by the environment have the implicit function. In mathematics, an implicit equation is a relation of the form *R*(*x*_1_, …, *x_n_*) = 0, where *R* is a function of several variables (often a polynomial), for example, *x*^2^ + *y*^2^ − 1 = 0. While, a distinctive function is a relation of the form *y* = *f*(*x*), where *y* can be described directly by equation containing independent variables, for example, *y* = *sin*(*x*). Therefore, if a value is corrected, other values may be corrected accordingly.

For example, if Nodes *I* and *J* are two separate nodes. At some time point, *t*, they measure the raw values *I_t_* and *J_t_*. If no object has passed through Node *I*, *I_t_* can be compared to *d*_0_—the error in this point is *E_It_* = (*d_0_* − *I_t_*). Further, if over some time interval, Node *I* and Node *J* measure at three times points *t*_1_, *t*_2_ and *t*_3_ obtaining two sets of raw data, {*I*_1_, *I*_2_, *I*_3_} and {*J*_1_, *J*_2_, *J*_3_} with errors of {*E_I_*_1_, *E_I_*_2_, *E_I_*_3_} and {*E_J_*_1_, *E_J_*_2_, *E_J_*_3_}, (*Err*(*I*) and *Err*(*J*)). Although *I*_1_ has no direct relation with *I*_2_ and *J*_1_, the environments are very similar for both of nodes at the time points, *t_1_*, *t*_2_ and *t*_3_. The errors caused by the environment have the inner relations that can be concluded from the trends of *Err*(*I*) and *Err*(*J*) over time, *i.e.*, *Err*(*J*) = *Fun*(*Err*(*I*)). If *I*_1_ is in Z-MODE, *E_I_*_1_ can be calculated directly, then *E_J_*_1_ can be calculated from the function, and *J*_1_ can then be found.

### 3.2. Models of NEC

In the air with a constant pressure, *v_s_* (shown in Equation 1) is given by the equation [27]:

(2) $$v_{s}=\sqrt{\frac{\gamma RT}{M}}$$

where *R* is the gas universal constant, *γ* is the specific heat ratio-the ratio of gas pressure fixing specific heat capacity in specific heat at constant volume, and *M* is the molar mass of the gas. The three variables all have minute changes in common [28]. *T* is the thermodynamic temperature of the gas (in Kelvin), which can be calculated from the temperature in centigrade *t_c_* by *T* = *c*_0_ + *t_c_*. *c*_0_ is the Absolute Temperature, a constant. Using Equation 2, it can be seen that the ultrasonic velocity will increase by approximately 0.6 m/s if the temperature increases every 1 °C. If the default velocity is around 340 m/s at 20 °C it is then the primary origin of measurement error in the UDS.

In addition, *v_s_* is also influenced by other factors of the environment, such as humidity and atmospheric pressure. As for Δ*T*, it also has unavoidable errors, arising from the attenuation of the ultrasonic waves and the features of the detected objects.

If *v_s_* has the error of *o*(*v_s_*) and Δ*T* has the error of *o*(Δ*T*), then the corrected *d_s_* (as *d̂_s_*) can be given by:

(3) $$\begin{array}{ll} {\hat{d}}_{s} & =\frac{\left(v_{s}+o(v_{s}))\times (\Delta T+o(\Delta T)\right)}{2}\\ & =\frac{v_{s}\times \Delta T}{2}+\frac{o(\Delta T)}{2}\times v_{s}+\frac{o(v_{s})}{2}\times \Delta T+\frac{o(v_{s})\times o(\Delta T)}{2} \end{array}$$

where, *o*(Δ*T*) × *v_s_* and *o*(*v_s_)* × Δ*T* are relations; it can be represented as:

(4) $${\hat{d}}_{s}=d_{s}+\text{err}(\alpha ,\beta ,\delta )$$

in which $d_{s}=\frac{v_{s}\times \Delta T}{2}$ and $\text{err}(\alpha ,\beta ,\delta )=\frac{o(\Delta T)}{2}\times v_{s}+\frac{o(v_{s})}{2}\times \Delta T+\frac{o(v_{s})\times o(\Delta T)}{2}=\alpha +\beta +\delta$. Considering that both *o*(*v_s_*) and *o*(Δ*T*) are infinitesimally small, and the infinitesimal order of *δ* is lower than *α* and *β*, the value of *err*(*α*, *β*, *δ*) heavily relies on *α* and *β*. Equation 3 also demonstrates that there is heavy coupling between *v_s_* and Δ*T* in both *α* and *β*. In order to decouple *v_s_* and Δ*T*, we decided to train the equation of *err*(*α*, *β*, *δ*) through sufficient experimental data fitting, using statistical algorithms.

Mathematical statistics has been widely used in the collection, analysis, interpretation and presentation of data in the process of error correction. Variance is used as a measure of discreteness. A set of numbers has a probability distribution, expressing how far the numbers deviate from *E* [*X*]. Variance is a parameter to describe either the actual probability distribution of an observed population of numbers or the theoretical probability distribution of a sample (a not-fully-observed population) of numbers. If a variable *X* has the expected value (mean), *μ* = *E* [*X*], then the variance of *X* is given by:

(5) $$\sigma^{2}=\text{Var}(X)=E\left[{\left(X-\mu \right)}^{2}\right]=E\left[X^{2}\right]-{\left(E[X]\right)}^{2}$$

where *σ*^2^ is the variance.

For two variables, covariance is a measure of the probability that they change together. The sign of the covariance shows the tendency in the linear relationship between the variables, and the magnitude of the covariance shows the strength of the linear relation. We define the covariance of the two variances *X* and *Y* as *Cov*(*X*, *Y*):

(6) Cov(X,Y)=E[(X−E[X])(Y−E[Y])]=E[XY]−E[X]E[Y]

While the magnitude of *Cov*(*X*, *Y*) is dependent on the magnitude of *X* and *Y*, it cannot compare the linear relationships between different variables, so the Pearson correlation coefficient (*ρ*) is defined to measure the correlation (linear dependence) between two variables, *X* and *Y*, giving a value on the interval [−1, 1]. If *Var*(*X*) ≠ 0 and *Var*(*Y*) ≠ 0, *ρ* is defined as:

(7) $$\rho (X,Y)=\frac{\text{Cov}(x,y)}{\sqrt{\text{Var}(X)}\sqrt{\text{Var}(Y)}}$$

Considering that *ρ*(*X, Y*) represents the relationship between *X* and *Y*, *ρ* can be used to quantize the relationship between an arbitrary node and its neighboring nodes. Data of some nodes can be corrected by data of others after *ρ*(*u, v*) has been obtained, because the relationship of two nodes *u* and *v*, chosen at random, is known.

An abstract model is set up to illustrate the problem. Assume all of the UDS distributed in a large area, form a matrix and are at time *t*; each of them can measure a value, *a_ij_*, in the position of *i* and *j*; then, *A* is the raw value matrix

(8) $$A_{t}=\left[\begin{array}{cccc} a_{00,} & a_{01}, & \cdots , & a_{0n}\\ a_{10,} & a_{11}, & \cdots , & a_{1n}\\ \cdots , & \cdots , & \cdots , & \cdots \\ a_{m0}, & a_{m1}, & \cdots , & a_{mn} \end{array}\right]$$

Some of these values could have been corrected by other methods, such as the Z-MODE of a UDS device. Assume that $\bar{a_{ij}}$ is the corrected value in the position of *i* and *j*, then *B′* is the corrected value array. For example, if *a*_00_, *a*_01_, *a*_11_, *a_mn_* are corrected to $\bar{a_{00}}$, $\bar{a_{01}}$, $\bar{a_{11}}$, $\bar{a_{mn}}$, then *B′* has four elements, $B^{\prime }=\left\{b_{0},b_{1},b_{2},b_{3}\right\}=\left\{\bar{a_{00}},\bar{a_{01}},\bar{a_{11}},\bar{a_{mn}}\right\}$. It can also be expressed as a sparse matrix relation: in a positron matrix *S* and value matrix is *B*:

(9) $$S_{t}=\left[\begin{array}{llll} 1, & 1, & \ldots , & 0\\ 0, & 1, & \ldots , & 0\\ \ldots , & \ldots , & \ldots , & \ldots \\ 0, & 0, & \ldots , & 1 \end{array}\right]$$

(10) $$B_{t}=\left[\begin{array}{llll} \bar{a_{00},} & \bar{a_{01},} & \ldots , & a_{0n}\\ a_{10}, & \bar{a_{11}}, & \ldots , & a_{1n}\\ \ldots , & \ldots , & \ldots , & \ldots \\ a_{m0}, & a_{m1}, & \ldots , & \bar{a_{mn}} \end{array}\right]$$

If *P* = 1 (each value in the matrix is one), then *B* = *B*_0_; it is an initial state. Using simple subtraction, the error matrix is easily obtained:

(11) $$Err_{t}=\left[\begin{array}{cccc} \bar{a_{00}}-a_{00}, & \bar{a_{01}}-a_{01}, & \ldots , & 0\\ 0, & \bar{a_{11}}-a_{11}, & \ldots , & 0\\ \ldots , & \ldots , & \ldots , & \ldots \\ 0, & 0, & 0, & \bar{a_{mn}}-a_{mn} \end{array}\right]$$

In this paper, the key information is that *a_ij_* is correlated with its neighboring nodes, which may be “one-to-one” or “one-to-many” relations. We define the neighbor array:

(12) $$\begin{array}{ll} N & =\{n_{0},n_{1},n_{2},\cdots \}\\ & =\{a_{\left(i-1)(j-1\right)},a_{\left(i-1)(j\right)},a_{\left(i-1)(j+1\right)},a_{\left(i)(j-1\right)},a_{\left(i)(j+1\right)},\cdots ,a_{\left(i+1)(j+1\right)}\} \end{array}$$

For *a_ij_*, if a correct function, *Cor_ij_*(*N*), has been found from the analysis of the history data, then the corrected *a_ij_* $\hat{a_{ij}}$ is

(13) $$\hat{a_{ij}}=Cor_{ij}(N)$$

Note that each *a_ij_* has its own correction function, *Cor_ij_*(*N*), and the function may also be dynamically corrected along with the accumulation of the history data. After the correction, the matrix *B* will be converted to a corrected matrix *C*:

(14) $$C_{t}=\left[\begin{array}{llll} \hat{a_{00}}, & \hat{a_{01}}, & \ldots , & \hat{a_{0n}}\\ \hat{a_{10}}, & \hat{a_{11}}, & \ldots , & \hat{a_{1n}}\\ \ldots , & \ldots , & \ldots , & \ldots ,\\ \hat{a_{m0}}, & \hat{a_{m1}}, & \ldots , & \hat{a_{mn}} \end{array}\right]$$

According to the analysis above, the key problem is to find the suitable *Cor_ij_*(*N*) for each *a_ij_*.

### 3.3. Relations between Neighboring Nodes

Neighboring nodes are located in fixed static positions and have similar trends with environment changes, so they may have some relations. As shown in Equation 7, *ρ* is defined as a measure of the correlation, which gives an indication of the strength of the linear relationship between the two random variables. If *ρ* = 0, then *X* and *Y* are uncorrelated, and the stronger the correlation, the closer |*ρ*| is to one.

As a simple case, in a time interval, [*t*_1_, *t*_2_], where each node of *n* nodes has collected 1,000 raw measured values. As mentioned above, the mathematical expectation matrix of the rough data of each node is *E_i_*

(15) $$E_{i}=\left[\begin{array}{c} \bar{a_{0}}\\ \bar{a_{1}}\\ \ldots \\ \bar{a_{n-1}} \end{array}\right]$$

where *i* = 0, 1, 2, ….

From Equations 6 and 7, the Pearson Correlation Coefficient (*ρ*) matrix is defined as *ρ_i,j_*.

(16) $$\rho_{i,j}=\left[\begin{array}{ccccc} 1 & \rho_{0,1} & \rho_{0,2} & \cdots & \rho_{0,n-1}\\ \rho_{1,0} & 1 & \rho_{1,2} & \cdots & \rho_{1,n-1}\\ \rho_{2,0} & \rho_{2,1} & 1 & \cdots & \rho_{2,n-1}\\ \cdots & \cdots & \cdots & \cdots & \cdots \\ \rho_{n-1,0} & \rho_{n-1,1} & \rho_{n-1,2} & \cdots & 1 \end{array}\right]$$

where *i, j* = 0, 1, 2, …

Of particular note is that the sum of each line in the *ρ_i,j_* matrix is not likely to be 1. A well-understood method of normalization is used to make the unsatisfactory *ρ_i,j_* matrix easier to use. Each value along the main diagonal is set to 0 each line standardized. Thus, a more useful matrix, *P*, is built:

(17) $$P_{i,j}=\left[\begin{array}{ccccc} 0 & P_{0,1} & P_{0,2} & \ldots & P_{0,n-1}\\ P_{1,0} & 0 & P_{1,2} & \ldots & P_{1,n-1}\\ P_{2,0} & P_{2,1} & 0 & \ldots & P_{2,n-1}\\ \ldots & \ldots & \ldots & \ldots & \ldots \\ P_{n-1,0} & P_{n-1,1} & P_{n-1,2} & \ldots & 0 \end{array}\right]$$

where *i, j* = 0, 1, 2, …

Matrix multiplication between *Err_t_* and *P_i,j_* brings a matrix of amended values, defined as the $\bar{Err_{t}}$ matrix. The corrected matrix, *C*, is determined by the simple combination of the rough value matrix, *A*, and the amended value matrix, $\bar{Err_{t}}$, which can be simply represented by the following equation: $C=A+\bar{Err_{t}}$.

### 3.4. Process of Network Error Correction

Based on the NEC model, we can assume that the measured data of *Sensor_i_* and *Sensor_j_* are *x_i_* and *x_j_*, respectively, which both follow Gaussian distribution. Their density functions, *P_i_*(*x*) and *P_j_*(*x*), respectively, can be used for the performance description of the sensor arrays. *x_i_* and *x_j_* are observed values of *X_i_* and *X_j_*, respectively.

**Figure 3.**
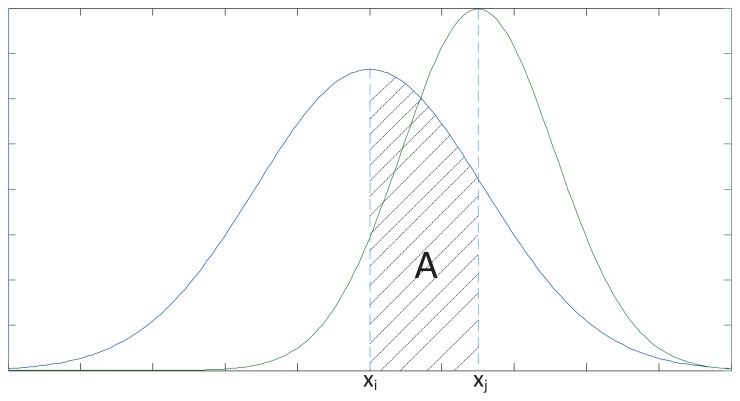
Schematic diagram of the confidence distance measurement.

We introduce fiducial distance measurement, in order to reflect the size of the error between *x_i_* and *x_j_*:

(18) $$Cor_{ij}^{2}\int_{j}^{i}p_{i}(x|x_{i})d_{x}=2A$$

where

(19) $$p_{i}(x|x_{i})=\frac{1}{\sqrt{2\pi }\sigma_{i}}\exp\left\{-\frac{1}{2}{\left[\frac{x-x_{i}}{\sigma_{i}}\right]}^{2}\right\}$$

and *A* is the area under the density probability plot *p_i_*(*x*|*x_i_*) over the interval, (*x_i_*, *x_j_*), as shown in Figure 3.

As mentioned above, *Cor_ij_* is defined as the fiducial distance measurement between the values of *Sensor_i_* and *Sensor_j_*, where 0 ≤ *d_ij_* ≤ 1. The smaller *d_ij_* the more similar the data *Sensor_i_* and *Sensor_j_* are, which means that *Cor_ij_* describes the degree of fusion between *Sensor_i_* and *Sensor_j_*.

*Cor_ij_* can be calculated by error function, *erf* (*θ*), which is:

(20) $$Cor_{ij}=\text{erf}\left(\frac{x_{j}-x_{i}}{\sqrt{2}\sigma_{i}}\right).$$

In the Learning Mode, three thresholds of *Cor_ij_*—namely *ε*_2_, *ε* and *ε*_1_—can be obtained after repeated trials. Then:

(21) $$\rho =\left[\begin{array}{ccccc} 1 & \rho_{0,1} & \rho_{0,2} & \ldots & \rho_{0,n-1}\\ \rho_{1,0} & 1 & \rho_{1,2} & \ldots & \rho_{1,n-1}\\ \rho_{2,0} & \rho_{2,1} & 1 & \ldots & \rho_{2,n-1}\\ \ldots & \ldots & \ldots & \ldots & \ldots \\ \rho_{n-1,0} & \rho_{n-1,1} & \rho_{n-1,2} & \ldots & 1 \end{array}\right]$$

where:

(22) $$\rho_{ij}=\{\begin{array}{cc} 0, & d_{ij}>{ɛ}_{2}\\ \frac{1}{2}-\frac{1}{2}\left(\frac{d_{ij}-ɛ}{{ɛ}_{2}-ɛ}\right), & ɛ<d_{ij}<{ɛ}_{2}\\ \frac{1}{2}, & d_{ij}=ɛ\\ \frac{1}{2}-\frac{1}{2}\left(\frac{d_{ij}-ɛ}{ɛ-{ɛ}_{1}}\right), & {ɛ}_{1}<d_{ij}<ɛ\\ 1, & d_{ij}\leq {ɛ}_{1} \end{array}$$

Subsequently, the standardized relationship matrix, *P_ij_*, can be acquired in the standardization process:

(23) $$p_{ij}=\{\begin{array}{cc} 0, & i=j\\ \frac{\rho_{ij}}{\Sigma_{j=0,j\neq i^{p_{ij}}}^{p_{ij}}} & i\neq j \end{array}$$

The data from one sensor are labeled as valid if the relationship value in the standardized relationship matrix *P_ij_* is large enough, which means that this sensor is supported by enough other sensors. Otherwise, if one sensor is supported by few or no sensors, its data are labeled invalid, which means that the data should be deleted. The set of all the valid data is called the fusion set *L* = {*x*_1_, *x*_2_, …, *x_l_*}, where the number of elements is called the optimum fusion number, *l*.

Based on the relationship matrix and fusion set, the maximum likelihood method has been adopted as the data fusion method. If

(24) $$p_{i}(x_{i}|\theta )=\frac{1}{\sqrt{2\pi }\sigma_{i}}\exp\left\{-\frac{1}{2}{\left[\frac{x_{i}-\theta }{\sigma_{i}}\right]}^{2}\right\}$$

where *i* = 1, 2, …, *l*, then:

(25) $$L(x_{1},x_{2},\ldots ,x_{l};\theta )=\prod_{t=1}^{l}p_{t}(x_{t}|\theta )$$

where *L*(*x*_1_, *x*_2_, …, *x_l_*;*θ*) is the maximum likelihood function based on the Maximum Likelihood Estimation (MLE), which means:

(26) $$\frac{\partial }{\partial \theta }L(x_{1},x_{2},\ldots ,x_{l};\theta )=\hat{\theta }=0$$

and the solution is:

(27) $$\hat{\theta }=\frac{\sum_{i=1}^{l}\frac{x_{i}}{\sigma_{i}}}{\sum_{i=1}^{l}\frac{1}{\sigma_{i}}}$$

where *θ̂* is the optimal fusion data of the fusion set, *L* = {*x*_1_, *x*_2_, …, *x_l_*}.

### 3.5. Description of the Two-Step Error Correction Process

#### 3.5.1. Previous Networked Error Correction Methods

**Figure 4.**
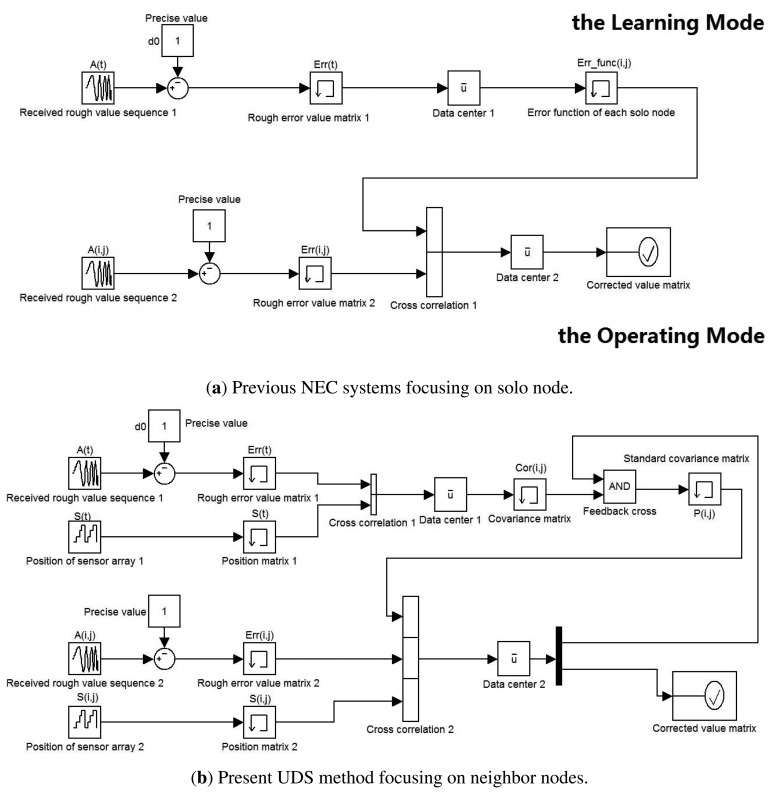
Design comparison and system improvements based on the previous NEC studies.

As mentioned above, the focus of the study is a novel method of error correction that lends weight to the argument that the accuracy of the UDS system relies heavily on the fitness and robustness of the function involving the association between the various patterns of the error, the position of the nodes, and the real-time temperature. Particularly noteworthy are previous studies on the NEC which, in general, consist of a Learning Mode and an Operating Mode, as shown in Figure 4a. The Learning Mode trains the error functions of the best fit by gathering a large number of rough data for a sufficiently long time. Consequently, the Operating Mode will deal with the real data gathered from the sensors in an industrial environment, in order to reduce the detrimental effect from the inevitable interference and to obtain a better response within the tolerable error. Compared with the previous methods, there are some significant improvements in the present work, as detailed in the following Section.

#### 3.5.2. Design Comparison and System Improvements

In Figure 4, it shows previous NEC systems and the present UDS method. It also illustrate the design comparison and the system improvements. We exploit not only the time domain model processing for each solo node, but also the relation matrix of neighboring nodes. Consequently, the present NEC system has three main advantages over the previous studies:

- Accuracy: instead of single-ended input, the way of input the present NEC system has adopted is differential input, which reduces measurement error, such as cosine error and Abbe error.
- Robustness: if one node is broken, previous NEC systems have no choice but to abandon its measured value, because the value is intolerant. However, the present NEC system can rationally estimate the value on the basis of the updated relation matrix.
- Renewability: in previous NEC systems, once the Learning Mode is over, the function of each node is be irrevocable, unless starting the Learning Mode over again. However, as shown in Figure 4b, the relation matrix is be updated through feedback cross-talk every measurement period, which means that there is no boundary between the Learning Mode and the Operating Mode. Consequently, when the environment has changed, for example, from noon to midnight, the present NEC system can automatically update the relation matrix in order to adapt to the external influence.

## 4. System Design of UDS

### 4.1. Design of the Sensor

The UDS is composed of three galvanically isolated main parts: a power module, a power amplifier module and a transmitter-receiver module. The power module provides two main functions: driving the sensors and ground protection. The power amplifier module, which is composed of a power amplifier and an inverting amplifier, can be integrated into a differential amplifier. Compared with the single-ended signal and the common-mode signal, the different-mode signal provides the following advantages:

- A small signal can be more easily detected by means of controlling a reference voltage.
- Since one specific interference source homogeneously affects both sides of the differential signal to a large extent, the different-mode signal is virtually immune to electro-magnetic interference.
- Because the different-mode system does not need to build a Virtual Ground at any point between ground and power when processing a bipolar signal in a single-supply system, the fidelity of the signal is much better than the single-ended signal and the common-mode signal.

**Figure 5.**
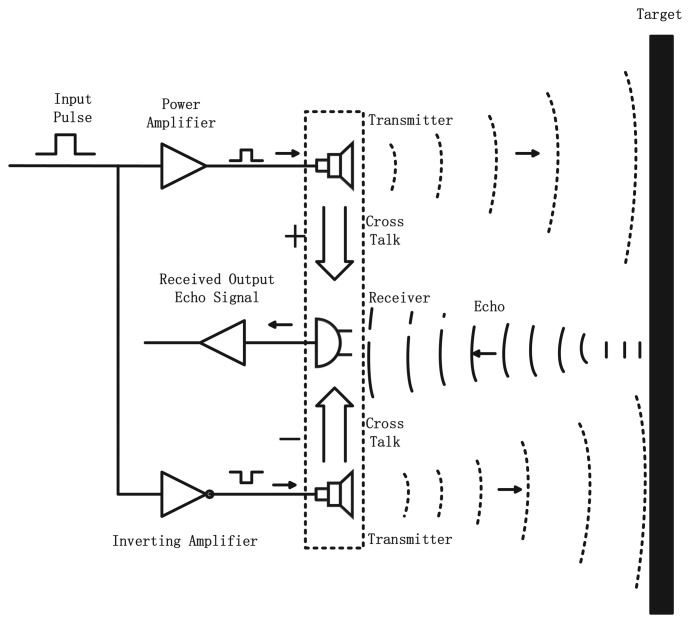
View of the design of the UDS.

The transmitter-receiver module is the most essential part of the application of the signal transduction. In the transmitter mode, the transmitters generate an ultrasonic beam; after the ultrasonic beam hits the target and rebounds, the addition of the two waveforms gives the return echo. In the receiver mode, the receiver acts as a mechanical detector of the reflected wave and outputs the echo signal already received. In Figure 5, it presents a view of the design of the UDS.

### 4.2. Architecture of the Sensor Arrays

Data of characteristic parameters measured by different sensors, even of the same type, are different to some degree. This deviation has two main causes: (1) sensor accuracy and (2) the mathematical algorithm adopted in data processing. The most important parameter in the UDS system is the distance data measured by the sensors, so we apply the same index parameter to multi-sensor measurement for the following reasons:

- Using many sensors with different performance and accuracy can complement each other's advantages and cover disadvantages.
- The redundancy configuration of sensors can improve reliability and measurement accuracy.

**Figure 6.**
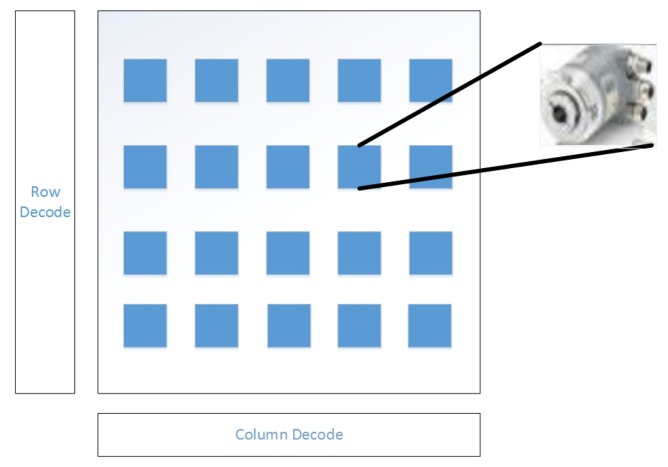
Architecture of the sensor arrays.

In Figure 6, it represents the architecture of the sensor arrays. Assuming that all the sensors are arranged as n-by-n matrices, log_2_*n* signals are needed for row decoding and log_2_*n* are for column decoding. Therefore, 2 log_2_*n* signals are all that is needed for locating any sensor at any position. There are two types of data the sensor arrays will transmit to the DC during data processing: the position signal and measured data. After cross communication between the row decoded signals and column decoded signals, the one-to-one relationship between the position of any sensor and its measured data can be established.

### 4.3. Interconnection of the Sensor Arrays

#### 4.3.1. Schematic of the Process of Selecting An Element

**Figure 7.**
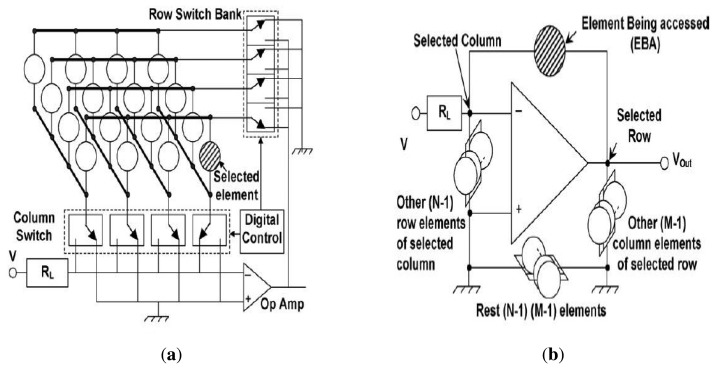
The equivalent reduced circuit when an element (EBA) is selected. (**a**) Proposed connection scheme with all the sensors having one end at a row line and another end at a column line. (**b**) Schematic of the proposed circuit showing the EBA filled with hashed lines.

The proposed circuit for a 4 × 4 sensor array is represented in Figure 7a for illustration of the scheme. However, the analysis is applicable to any *N* × *M* sensor array As shown in the Figure 7a, the columns and rows are connected to two digitally controlled single pole double throw switch banks, which allows connecting of any column to the load resistor, *R_L_*, and any row to the output node of the operational amplifier, with all other rows and columns staying connected to the ground. The element being accessed comes in the negative feedback path of the Operational Amplifier (Op Amp), as represented in Figure 7b. The other *N* − 1 sensors connected to the selected column make a parallel combination across the two inputs of the Op Amp, with non-inverting inputs connected to the ground. Similarly, *M* − 1 elements connected to the selected row get connected to the output node of the Op Amp with their other end grounded. The rest of the (*N* − 1) × (*M* − 1) elements, not physically connected to the Elements Being Accessed (EBA), have both of their ends at the ground [29].

#### 4.3.2. Neighbor Discovery Algorithm

The senor network can be modeled as a weighted directed graph (*G*), of which the nodes belong to the point set (*V*), while the network communication links belong to the edge set (*E*(*G*)). The communication link between Node *u* and Node *v* is defined as *e*(*u, v*), whose value of weight is defined as the data packet acceptance rate. According to the length of the forward path, we have defined four types of neighbor nodes:

(1) The theoretical neighbor of Node *v*: (*N*(*v*)) :
  {*u*|*u* ∈ *V*(*G*) ∧ *e*(*v, u*) ∈ *E*(*G*)}.
(2) The non-forward neighbor of Node *v*: (*N*_0_(*v*)) :
  {*u*|*u* ∈ *V*(*G*) ∧ *e*(*v, u*) ∈ *E*(*G*) ∧ *e*(*u, v*)}.
(3) The one-step-forward neighbor of Node *v*:
  (*N*_1_(*v*)) : {*u*|(*u* ∈ *N*_0_(*v*)) ∨ (*u* ∈ *V*(*G*) ∧ *e*(*v, u*) ∈ *E*(*G*) ∧ ∃*m* ∈ *V*(*G*) ∧ *e*(*u, m*), *e*(*m, v*) ∈ *E*(*G*) ∧ *e*(*u, v*))}.
(4) The two-steps-forward neighbor of Node *v*:(*N*_2_(*v*)) :
  {*u*|(*u* ∈ *N*_1_(*v*)) ∨ (*u* ∈ *V*(*G*) ∧ *e*(*v, u*) ∈ *E*(*G*)∧
  ∃*m, n* ∈ *V*(*G*) ∧ *e*(*u, m*), *e*(*m, n*), *e*(*n, v*) ∈ *E*(*G*) ∧ *e*(*u, v*))}.

Under the precondition that the topological structure of the wireless sensor network is known, we can obtain the neighborhood discovery algorithm of the UDS as shown in Algorithm 1.



**Algorithm 1** Procedure *Calc* – *in* – *onb*(*G*, *N*_0_, *N*_1_, *N*_2_, *IN*_1_, *IN*_2_)
**Input:**
*G*;**Output:**
*N*_0_; *N*_1_; *N*_2_; *IN*_1_: The one-step-forwarding neighbor of Node *v; IN*_2_: The two-step-forwarding neighbor of Node *v*;1:**for all**
*v* ∈ *V* (*G*) **do**2: *N*_0_(*v*), *N*_1_(*v*), *N*_2_(*v*) ← *φ*3: **for all**
*e*(*u*, *v*) ∈ *E*(*G*) **do**4:  **if**
*e*(*u*, *v*) ∈ *E*(*G*) **then**5:   *N*_0_(*v*), *N*_1_(*v*), *N*_2_(*v*) ← *u*6:**else if** ∃*m* ∈ *V*(*G*) ∧ ∃*e*(*u, m*), *e*(*m*, *v*) ∈ *E*(*G*) **then**7:   *N*_1_(*v*), *N*_2_(*V*) ← *u*8:  **else if** ∃*m*, *n* ∈ *V* (*G*) ∧ ∃*e*(*u*, *m*), *e*(*m*, *n*), *e*(*n*, *u*) ∈ *E*(*G*) **then**9:   *N*_2_(*v*) ← *u*10:  **end if**11: **end for**12:**end for**13:**for all**
*v* ∈ *V* (*G*) **do**14: *IN*_1_(*v*) ← *N*_1_(*v*) – *N*_0_(*v*)15: *IN*_2_(*v*) ← *N*_2_(*v*) – *N*_0_(*v*)16:**end for**


## 5. Experiments and Results

### 5.1. Comparison of Initial Data

In order to simplify the UDS system, the experiments are based on a sample of sensor arrays, represented as matrices on the order of twenty by twenty. It makes the comparison of initial data between the ideal response, response in the absence of noise, and response with signal noise. The height of the semi-finished products is 50 cm, so the default distance of the parallel lines with none of the products passing by in the workshop that we used for experiments is also 50 cm. Therefore, the ideal value matrix has elements with value either 50 cm or 100 cm. Considering that interference highly depends on the position of the sensors in this model, we have obtained the initial data.

**Figure 8.**
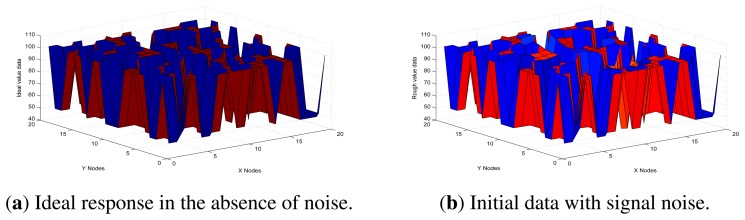
Comparison of initial data.

In Figure 8a, it shows the case of ideal response in the absence of noise, for which there are only two possible values, depending on which mode the sensor is in. This demonstrates that at a specific time, whether there is a semi-finished product passing by the sensor or not, the response is irregular. By comparing histograms in Figure 8a and Figure 8b, we can observe that there is macroscopic difference between the initial data and the ideal response, which means the measuring error is statistically significant. Furthermore, the mean absolute percentage error was 11.04%, which would not be totally acceptable in an industrial setting. Thus, in Figure 8, it shows that, in order to obtain a better performance, it is necessary to compensate errors resulting from the noise of the environment.

**Figure 9.**
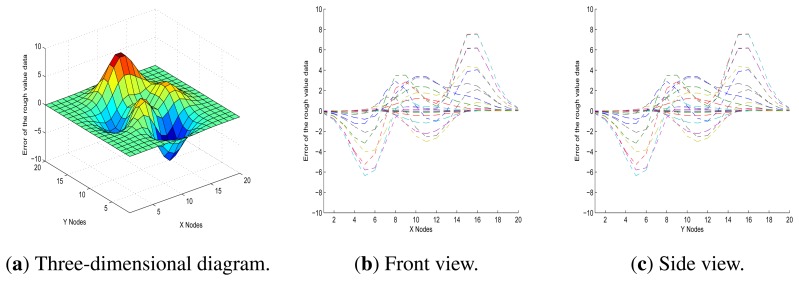
Initial data errors.

In Figure 9, it shows the front and side views of a three-dimensional representation of the initial data errors, which is a better visualisation than that in Figure 8, since it is in relative terms and demonstrates more clearly the association between measurement error and noise from the environment. Furthermore, in Figure 9, it also shows that there is little difference between the front view and the side view. One plausible explanation is that the error function graph of each node is approximately symmetrical with rotation around the perpendicular. Based on these factors, we decided to focus on the front view for this model.

### 5.2. Analysis of the Relationship between Nodes in Not Z-MODE and Nodes in Z-MODE

**Figure 10.**
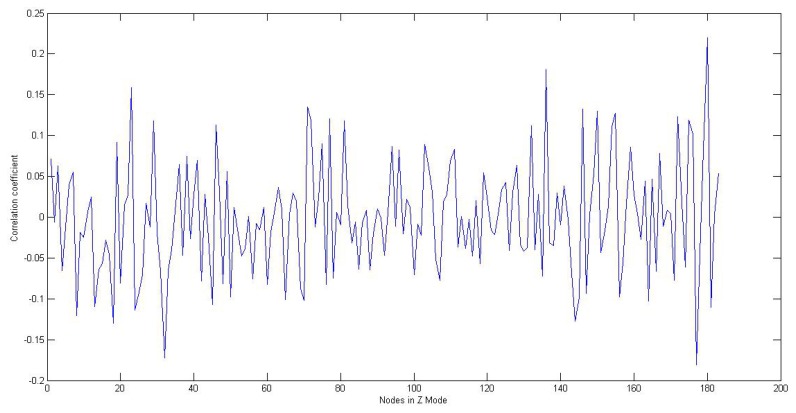
Correlation coefficient between a node in Not Z-MODE and the nodes in Z-MODE, obtained in the Learning Mode.

From Figure 10, we can observe that the correlation coefficient fluctuates a great deal with the change of the relative position between the node in Not Z-MODE and the nodes in Z-MODE. Although this observation cannot establish cause and effect, it strongly suggests that the size of interference is largely related to relative position. Additionally, one of the plausible interpretations of this observation, considering the field of a manufacturing workshop shown in Figure 1, is that the whole production process can be decomposed into several smaller, repeated processes, where interference from the environment in subprocess *I* is approximately parallel to subprocess *J*. This observation lends significant weight to the argument that it is reasonable for the value of an arbitrary node to be modified through a two-step process:

(1) Select a set of data for which the correlation coefficients are large enough.
(2) Correct this value of the arbitrary node based on the set data chosen in the first step.

### 5.3. Process of Error Correction

**Figure 11.**
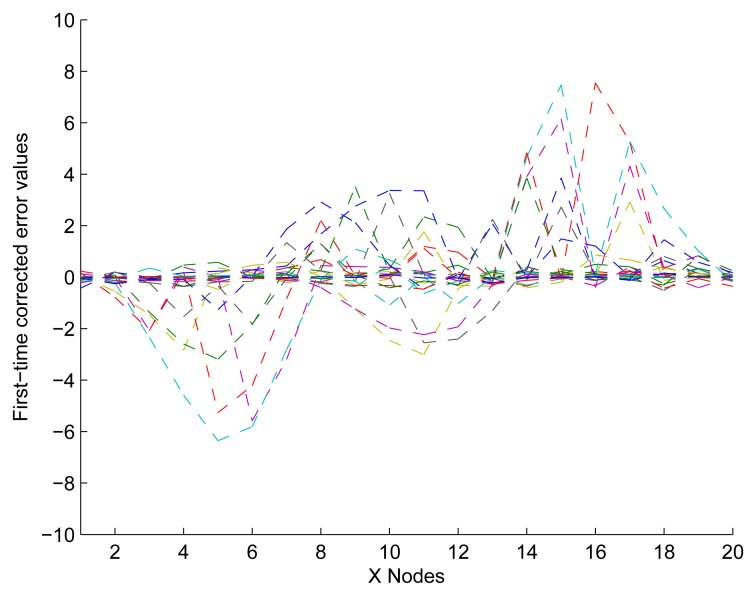
First-time corrected error values by applying a UDS.

In Figure 11, it shows that the experimental response is more accurate than the initial data shown in Figure 9. Although some nodes' values were close to zero, the others were undesirably large. Furthermore, the reason for this observation is that we have only modified the nodes in Not Z-MODE and not the nodes in Z-MODE. Consequently, additional optimization is required to obtain a desirable response.

**Figure 12.**
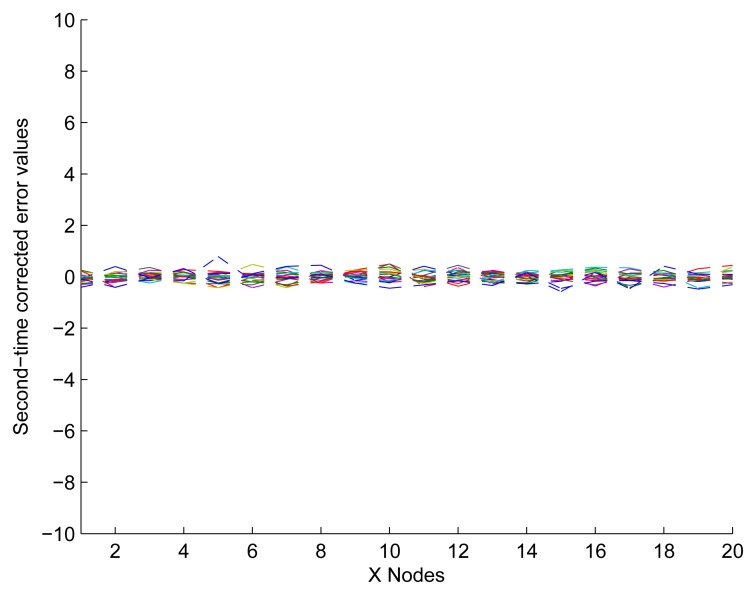
Second-time corrected values by applying a UDS.

In Figure 12, it shows that the experimental data have been fitted to ideally respond after modifying the nodes in Not Z-MODE. Compared with Figure 9b, the optimized method of distance measurement with the capability of NEC performs excellently, since it takes full advantage of the correlation coefficient among neighbor sensors. So far, we have described the process of one application to the UDS. However, determining a reasonable number of iterations is of great importance.

### 5.4. Optimal Number of Iterations

**Figure 13.**
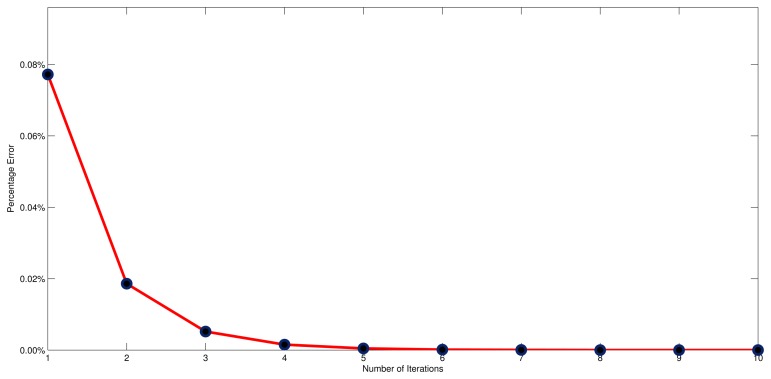
Mean absolute percentage error along with the increasing of iterations.

In Figure 13, it shows the tendency of the mean absolute percentage error along with the increasing of iterations, from which we can observe that the mean absolute percentage error has significantly reduced, from 0.08% to 0.01% in the first three iterations. This observation demonstrates that the adaptive NEC system has the ability to eliminate the steady state error. Moreover, the mean absolute percentage error uniformly converges to zero once the number of iterations is large enough, and the convergence rate is approximately O(ln). The reduced rate suddenly slows down from the fourth iteration, however. Taken together, this indicates that the most reasonable number of iterations is three, since this gives a desirable response without extra cost.

## 6. Conclusions

A novel method based on NEC for obtaining a satisfactory distance measurement has been proposed in this work. It can provide a high-speed and high-accuracy solution for distance measurement, which is vital in most industrial fields. One of the most obvious flaws of the previous methods is that none of them has considered the indirect association among neighboring sensors, which leads to less accuracy and extra cost. Therefore, one of the main aims of this study was to propose a novel method based on NEC that can provide a high-speed and high-accuracy solution for distance measurement. We applied the maximum likelihood method to determine the optimal fusion data set and used a neighbor discovery algorithm to identify neighboring nodes at high speed; furthermore, we adopted the NEC optimization algorithm, which takes full advantage of the correlation coefficient among neighboring sensors. The experimental results demonstrate that the ranging errors of the NEC system are within 2.20%, and the mean absolute percentage error is reduced to 0.01% after three iterations of this method.

Our proposed method has several strengths. However, several limitations merit comment. The most critical of them is that we are unable to establish and then test the exact cause and effect equation of the numerical error and the propagation of the ultrasonic wave affected by the environment, such as temperature, humidity and atmospheric pressure. Nevertheless, in spite of this limitation, we believe this study opens new paths of investigation in distance measurement using a UDS and, to some extent, modifies the manner in which we understand the method of error compensation, because new insights into it will likely generate novel methods to measure distance more effectively and quickly than previously. There is, therefore, a great need for further research in this area.
